# Single Alkali Metal Ion-Activated Porous Carbon With Heteroatom Doping for Supercapacitor Electrode

**DOI:** 10.3389/fchem.2020.00815

**Published:** 2020-09-15

**Authors:** Changshui Wang

**Affiliations:** Department of Radiochemistry, China Institute of Atomic Energy, Beijing, China

**Keywords:** microporous carbon, energy density, sulfur-doping, supercapacitor, alkali metal ion activation, specific capacitance

## Abstract

A single alkali metal ion activation method was used to prepare sulfur-doped microporous carbons. A series of alkali metal ions such as Li^+^, Na^+^, K^+^, and Cs^+^ was used in the polymerization process of 3-hydroxythiophenol and formaldehyde to obtain metal ion anchored in the sulfur-containing resin, which was further treated to obtain xerogel and carbonized to obtain microporous carbon with sulfur doping. In this case, the monodispersed alkali metal ions could realize highly effective activation with low activating agent dosage. Intensive material characterizations show that the alkali metal ions determine the pore structure and surface properties of as-prepared carbons. C-Cs prepared by Cs^+^ ion possesses a high Brunauer–Emmett–Teller specific surface area of 1,037 m^2^ g^−1^ with interconnected microporosity and sulfur doping. The specific capacitance of C-Cs can reach up to 270.9 F g^−1^ in a two-cell electrode measurement system, whereas C-Cs-based supercapacitors can deliver an energy density of 7.6 Wh kg^−1^, which is much larger than that of other samples due to its surface functionalities and well-interconnected porosities.

## Introduction

As a traditional electrode material for supercapacitors (Huang et al., 2015; Borenstein et al., 2017; Raza et al., 2018; Zhang et al., 2018; Han et al., 2019; Li et al., 2019), porous carbon has attracted attention from researchers due to its good electric conductivity, high specific surface area, adjustable porous structure, and surface functionalities (Bi et al., 2019; Deng et al., 2019; Wang et al., 2019; Huo et al., 2020; Li et al., 2020). Among those merits, the surface area for carbon plays a key role in the energy storage process, as the energy storage mechanism for porous carbon is electric double layer (EDL) capacitance-dominated, together with potential pseudocapacitance generated by heteroatom functionalities on the surface of porous carbon (Zhang and Zhao, 2009). The high surface area of porous carbon can provide more interface to accommodate electrolyte ions during the energy storage process. However, studies have shown that the capacitance does not increase linearly with surface area (Raymundo-Piñero et al., 2006; Ghosh and Lee, 2012). The areal capacitance (capacitance per unit surface area) for carbon material is limited below 25 μF cm^−2^ (Conway, 1999). Porous structures such as pore connectivity, pore size, and pore size distribution also have a significant effect on capacitance. The science of electrolyte ions' penetration in micropores during the charging process was disclosed in recent years. Gogotsi group found that there is an anomalous increase of capacitance at pore size <1 nm in carbide-derived carbons (Chmiola et al., 2006). Distortion of solvation shell and desolvation of ions in subnanometer pores were proposed when the solvated ions were charged under a potential. In this case, electrolyte ions with decreased solvation shell would remarkably decrease the distance between the ion center to the electrode surface, thus increasing the capacitance (Chmiola et al., 2006, 2008). As the pore size of the carbide-derived carbons could be a concisely controlled, electrochemical study on this type of carbon shows that high capacitance could be realized when the pore size is perfectly adapted to the ion size (Largeot et al., 2008). It is also showed that high capacitance could be achieved when the pore size of microporous carbon is approximately two times of KOH ions in aqueous electrolyte (Zhou et al., 2016). Therefore, microporous carbons with a pore size that adapted well with electrolytes are highly desirable for supercapacitors. Many researchers are focusing on the pore structure architecture of porous carbons. Traditional activation method using chemical or physical activating agents and soft or hard templating methods were intensively developed in recent years (Gaslain et al., 2006; Nishihara et al., 2009; Zapata-Benabithe et al., 2012; Kim et al., 2016; Li et al., 2016; Wu et al., 2017; Yang et al., 2019). However, there are still some inevitable drawbacks for the traditional activation method or templating method such as high activating agent dosage, low carbon yield, and complex operation procedure.

Apart from pore structure adjustment, surface heteroatom doping can bring about an obvious enhancement of supercapacitive performance for carbon materials, especially in aqueous electrolytes due to the following reasons: (i) surface functionalities can react with electrolyte ions to generate pseudocapacitance. Oxygen and nitrogen functionalities are believed to bring about pseudocapacitance by reacting with electrolyte, especially in an acid electrolyte. Numerous researches have been reported on the preparation of nitrogen- and oxygen-doped porous carbons with enhanced supercapacitive performance (Hulicova-Jurcakova et al., 2012; Zapata-Benabithe et al., 2012; Zhang et al., 2012). (ii) Some surface functionalities can even widen the potential window of aqueous electrolytes. Denisa group prepared a series of P-doped carbons or P-doped graphenes with a working potential window of 1.5, 1.6, and 1.7 V in aqueous electrolytes (Huang et al., 2013; Wen et al., 2015, 2016). The assembled supercapacitor working in such a high potential window could deliver two times higher energy density than that in a 1-V potential window. Such a widening potential window is attributed to the blockage of active oxidation sites by phosphate groups (Hulicova-Jurcakova et al., 2009). (iii) Surface functionalities can increase the hydrophilicity of porous carbon. As the introduction of polar functionalities, the hydrophilicity will be promoted accordingly, thus improving the wettability of carbon surface to accommodate more aqueous electrolyte ions even at high current densities (Kicinski et al., 2014; Zhang et al., 2014; Hasegawa et al., 2015).

Considering the earlier mentioned views of porous carbon, constructing a microporous carbon with developed porous structure and heteroatom doping can contribute to high-performance electrode materials for supercapacitors. Herein, we prepared a series of sulfur-doped microporous carbons with the precursor of 3-hydroxythiophenol and formaldehyde. A series of alkali metal ions such as Li^+^, Na^+^, K^+^, and Cs^+^ was used in the polymerization process of carbon precursors to obtain metal ion anchored in the sulfur-containing resin, which was further treated to obtain xerogel and carbonized to obtain microporous carbon with sulfur doping. In this case, the uniformly anchored alkali metal ions could realize effective activation with a low activating agent dosage. Intensive material characterizations show that the alkali metal ions determine the pore structure and surface properties of as-prepared carbons. C-Cs prepared by Cs^+^ ion possesses a high Brunauer–Emmett–Teller (BET) specific surface area of 1,037 m^2^ g^−1^ with interconnected microporosity and sulfur doping. The specific capacitance of C-Cs can reach up to 270.9 F g^−1^ in a two-cell electrode measurement system, whereas C-Cs based supercapacitors can deliver a high energy density of 7.6 Wh kg^−1^ due to its surface functionalities and well-interconnected porosities.

## Experimental Section

### Preparation of C-Ms

In a typical synthesis, 3-hydroxythiophenol (1 g, 7.9 mmol) was dissolved in 8-ml ethanol/H_2_O (volume ratio of 1:1); then, alkali hydroxide (8.7 mmol) and formaldehyde (37 wt%, 15.8 mmol) were added into the mentioned transparent solution under magnetic stirring. The solution was solidified at 80°C for 24 h. The resulting hydrogel was cooled down to room temperature and immersed in acetone for 12 h to exchange the water with acetone. The exchange operation repeats four times, and the gel was removed from acetone and naturally dried to obtain xerogel before further carbonization. The xerogels are labeled as X-M, where M represents for alkali metal ion. The xerogel was carbonized in a tube furnace under Ar atmosphere at a flow rate of 50 ml min^−1^. The sample was heated at 200°C for 1 h with a heating ramp of 2°C min^−1^ and then carbonized at 700°C for 1.5 h with a heating ramp of 5°C min^−1^. The obtained carbon samples were washed with diluted HCl and deionized water to neutrality. The samples are labeled as C-M, where M stands for the alkali metal ion.

### Characterization of C-Ms

The morphology of the samples was observed using a scanning electron microscope (SEM, FEI, Holland) and transmission electron microscopy (TEM, JEOL2010, Japan). The crystal structure of the C-Ms was determined by X-ray diffraction (X'Pert PRO MPD, PANalytical, Holland) using Cu Kα radiation (λ = 0.15418 nm) and Raman spectroscopy (JY Labram HR 800, France) using a 632.8-nm wavelength laser. The surface properties of C-Ms were characterized by X-ray energy dispersive spectroscopy (EDS, BRUKER AXS) and X-ray photoelectron spectroscopy (PHI5000VersaProbe, ULVAC-PHI, Japan). Nitrogen adsorption–desorption isotherms were measured at liquid nitrogen temperature (77 K) using a surface area and porosity analyzer (ASAP2020M, Micromeritics, USA). The carbon samples were degassed under a turbomolecular vacuum at 300°C for 6 h before sorption measurements. N_2_ gases with super high purity (99.999%) were used for the physisorption measurements. The BET equation was used to calculate the apparent surface area from N_2_ adsorption data. For advanced porosity analysis, pore size distributions, cumulative surface areas, and cumulative pore volumes were determined by using quenched solid-state density functional theory (QSDFT) method considering sorption of nitrogen at 77 K in carbon as a model adsorbant and slit-like pores as a pore model. The Quantachrome Autosorb ASiQwin 2.0 software supplied the implemented QSDFT model.

### Fabrication of Electrodes and Electrochemical Measurements

Working electrodes were made by pressing the electrode materials onto nickel foam, in which the electrode materials contained 5 wt% polytetrafluoroethylene as a binder. The mass of active materials in each working electrode is about 2 mg. To ensure that the active materials are thoroughly wetted with the electrolytes of 6-M potassium hydroxide, the working electrode was vacuum-impregnated with the electrolyte before electrochemical tests. The electrochemical capacitive performances of samples were studied on a CHI660E electrochemical workstation. All electrochemical measurements, including cyclic voltammetry (CV) and galvanostatic charge–discharge, were performed in a two-electrode system using two electrodes. A potential window of 0–0.9 V (6-M KOH electrolyte) was applied to the electrochemical measurements. The specific capacitance was calculated from the discharge curves according to the equation *C*_*m*_ = *4It/Vm*, where *C*_*m*_, *I, t, V*, and m are the specific capacitance, discharge current, discharge time, potential window, and the total mass of active materials in the cell, respectively. The energy density (*E*) and power density (*P*) of the cell could be calculated according to the equations: *E* = *C*_*m*_*V*^2^/(*8* × *3.6), P* = *2E*/*t*, where *C*_*m*_, *t*, and *V* are the specific capacitance, discharge time, and potential window in the cell, respectively.

## Results and Discussion

### Surface Property and Structure Analyses of C-Ms

**Figure 1:**
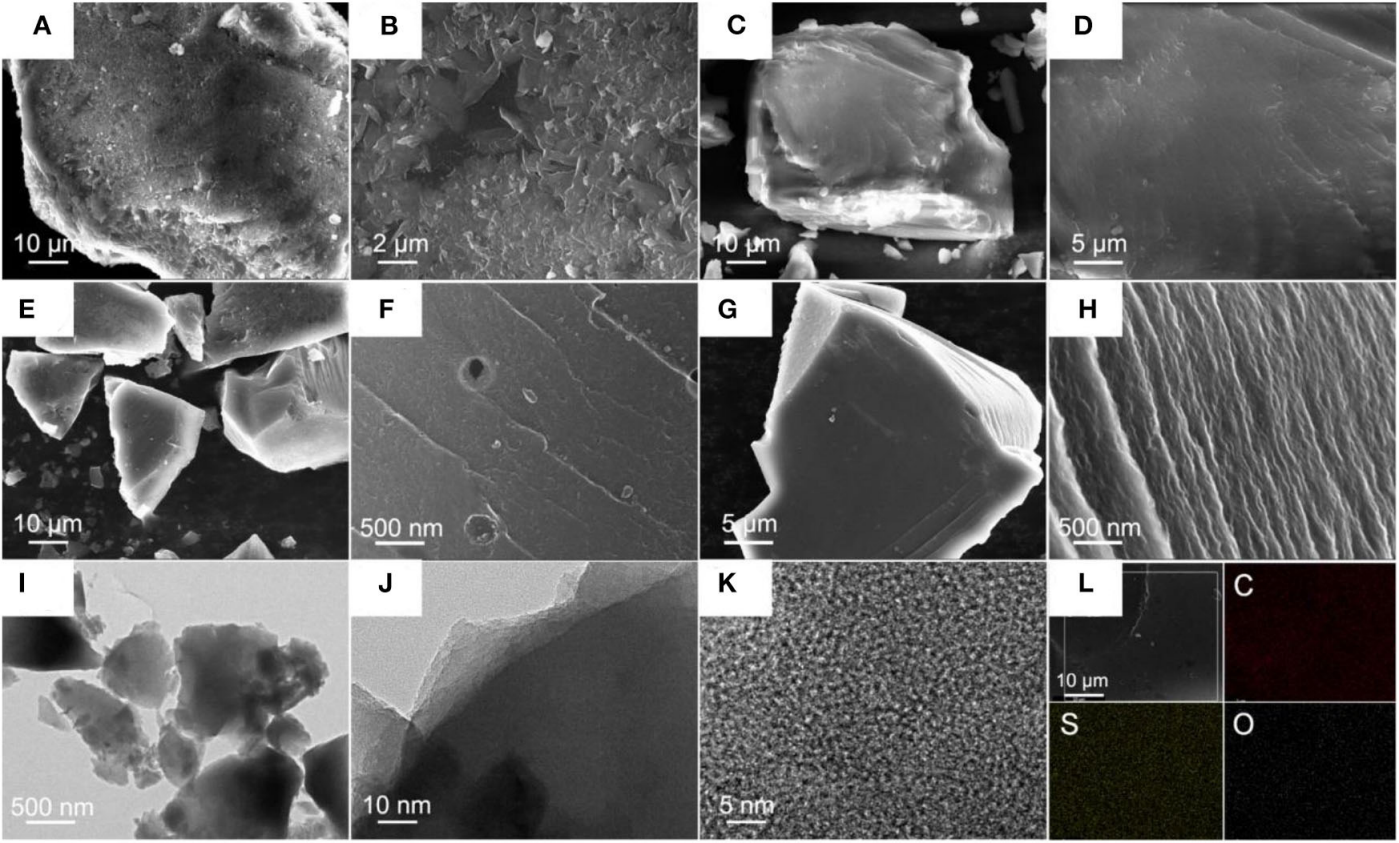
SEM images for X-Na **(A,B)**, X-K **(C,D)**, C-Na **(E,F)**, and C-Cs **(G,H)**; TEM images for C-Cs **(I**–**K)**; EDS mapping **(L)** for C-Cs.

SEM images of X-Na (Figures 1A,B) show that the bulk structure is composed of numerous flakes with the size of several macrons. Many stacking pores generated by those flakes could also be observed everywhere. By contrast, the bulk structure of X-K is composed of a large layered structure with no macropores on its surface (Figures 1C,D). The difference between those two xerogels reveals that the alkali metal ion has a key impact on the formation of resin. A metal ion with higher Lewis acidity led to form resin with a relatively low polymerization degree. As the xerogels are carbonized to C-Ms, the stacking pores in C-Na could be observed, whereas smooth surface in C-Cs is observed in their corresponding bulk structures (Figures 1E,G). The morphologies of the obtained carbons are similar to their corresponding carbon precursors, suggesting that the carbon precursors are quite stable during the carbonization process. Compared with C-Na (Figures 1E,F), there are no obvious macropore observations, smooth surface, and uniform wrinkle-like structures in C-Cs (Figures 1G,H); all reveal a more homogeneity for C-Cs. As confirmed by TEM observations (Figures 1I,J), there are no macropores in C-Cs as existed in C-Na (Figures 1E,F). Layered edge in Figure 1F can coincide well with SEM images, which suggests that the morphologies of xerogels are well reserved through the carbonization process. More importantly, although SEM images observe no macropores, numerous uniformed and developed micropores are found in the HR-TEM image for C-Cs (Figure 1K). Besides, no meso- or macropores are observed in Figure 1K. EDS mappings for C-Cs (Figure 1L) show that C, O, and S elements are uniformly distributed in C-Cs. No metal atoms were found in the EDS mapping, suggesting that the metal species can be completely removed by post-acid-washing. All of the pieces of evidence provided by SEM and TEM for C-Cs can confirm that alkali metal ions uniformly dispersed in the resin precursor could effectively realize the activation of xerogels.

**Figure 2:**
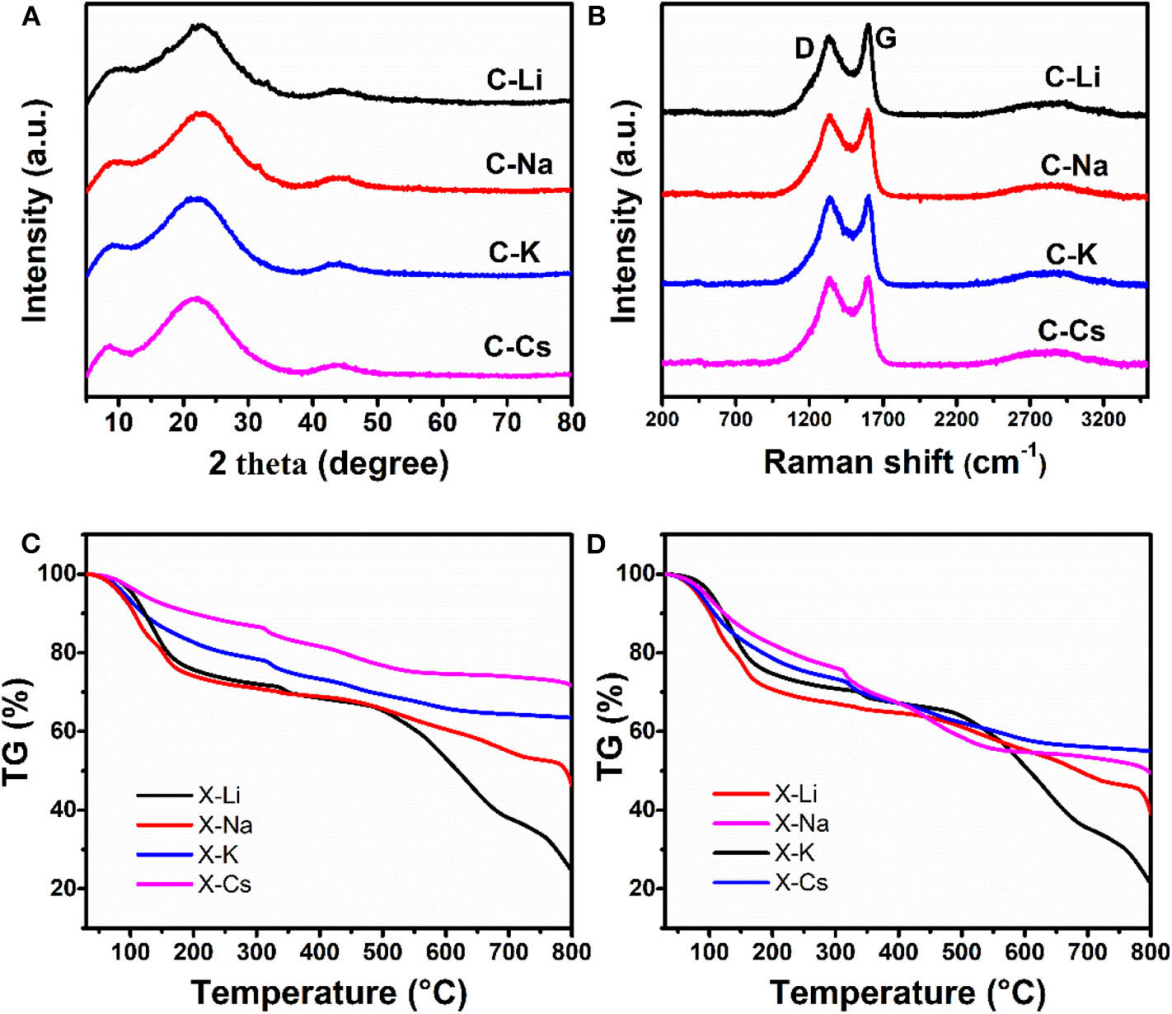
X-ray diffraction patterns **(A)**, Raman spectra **(B)** for C-Ms, TGA for X-Ms **(C)**, normalized TG curves derived from c as each X-M subtract the corresponding metal content **(D)**.

The microstructure for C-Ms is investigated by X-ray diffraction patterns and Raman spectra (Figures 2A,B). The diffraction peak centered at ~22° assigning to (002) lattice face, whereas a weak peak at 43° corresponding to the (100) lattice face is observed in all samples, indicating a turbostatic carbon structure for all samples. Two prominent peaks marked as D and G bands in the Raman spectra are observed for as-prepared carbons. The D band originates from the disorder-induced mode associated with structural defects and imperfections, whereas the G band corresponds to the first-order scattering of the E2g mode from the sp^2^ carbon domains (Park et al., 2011). The intensity ratios I_D_/I_G_ of C-Li, C-Na, C-K, and C-Cs are calculated to be 0.86, 0.95, 0.99, and 1, respectively, revealing an increase of disorder degree as the increasing of metal ion radius. Besides, the I_D_/I_G_ value could be used as a measure of the in-plane crystallite size of C sp^2^ domain in carbon samples. The increase of I_D_/I_G_ value as the increase of metal ion radius indicates a decreased in-plane crystallite size (CançAdo et al., 2006). This variation is probably due to the effective activation of xerogel by metal ions, thus destroying the in-plane crystallite structure by generating abundant micropores as confirmed by TEM and nitrogen sorption analysis. TG analysis of xerogels under the nitrogen atmosphere is used to study weight loss during the carbonization process (Figure 2C). Normalized TG curves where each metal content is subtracted from the corresponding xerogel-M samples are shown in Figure 2D. X-Na and X-Li have a much higher weight loss than that of X-Cs and X-K at the temperature range of 200–300°C and higher than 650°C (Figures 2C,D). This result can be probably attributed to the low polymerization degree of X-Na and X-Li, which will decompose under thermal treatment. By contrast, X-Cs and X-K with higher polymerization degrees show a better thermostability. The carbon yields for X-Cs and X-K at 750°C are about 55.6 and 52.3% (Figure 2D), respectively.

**Figure 3:**
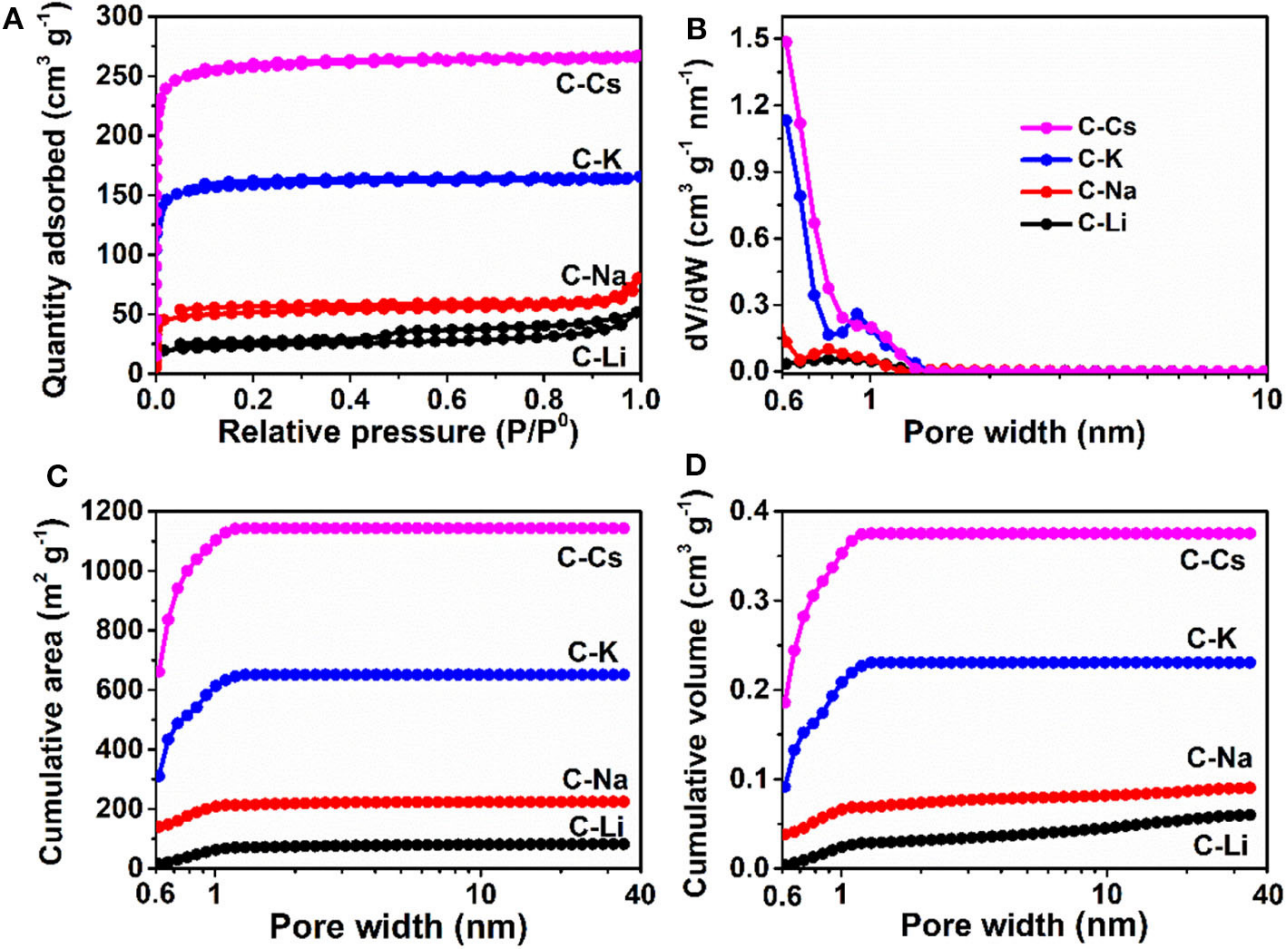
Nitrogen sorption isotherms **(A)**, pore size distribution **(B)**, cumulative surface area **(C)**, and cumulative pore volume **(D)** for C-Ms.

Nitrogen sorption analyses were used to explore the surface area and pore-structure parameters of the carbon materials. Nitrogen sorption isotherms of C-K and C-Cs (Figure 3A) are a typical type-I isotherm, where the adsorbed quantity rapidly increases as the dosage of nitrogen in the low relative pressure region (P/P^0^ < 0.1) and reaches a plateau afterward, indicating a micropores-dominated pore-structure for C-K and C-Cs. Isotherms for C-Li and C-Na show the same characteristic in the low relative pressure region (P/P^0^ < 0.45), suggesting the presence of micropores in these samples. However, the presence of a hysteresis loop in C-Li and the sharp increase of adsorbed quantities in the high relative pressure region in both samples suggest that there are numerous mesopores and macropores in C-Li and C-Na. The adsorbed quantity of plateau (P/P^0^ < 0.4) for each sample obeys the following order: C-Cs > C-K > C-Na > C-Li, suggesting that micropore content obeys the same order as the adsorbed quantity in this region is contributed from micropores. The BET surface areas and total pore volumes (Table 1) for C-Ms show the same order. C-Cs possesses the highest S_BET_ of 1,037 m^2^ g^−1^. The QSDFT pore size distributions (Figure 3B) for all samples show that substantial micropores are visible in these samples. As for C-Na, C-K, and C-Cs, pore widths are distributed within two regions (Figure 3B and Table 1): <0.7 and 0.7–1.3 nm. Although the former region does not show the peak value due to the restriction of adsorbate (Wu et al., 2014), the pore value in the latter region does vary as the change of alkali metal ions. The pore widths for C-Na, C-K, and C-Cs are 0.785, 0.926, and 1.007 nm, respectively. The increase of pore width is most probably due to the usage of alkali metal ions with different radius. The cumulative surface area and cumulative pore volume (Figures 3C,D) suggest that there are numerous micropores with a pore width of <0.6 nm. Both the cumulative surface areas and cumulative pore volumes contributed by the pore width of <0.6 nm for C-Ms obey the same order of C-Cs > C-K > C-Na > C-Li. The result could coincide well with nitrogen sorption isotherms. Such an order could be attributed to the enhanced reactivity of metal oxide with carbon. According to a previous study, alkali salt of resin can decompose into alkali carbonate, which will further decompose to the metal oxide, and the latter can react with carbon species under high temperature by forming numerous micropores. (Zhou et al., 2016)

**Table 1:** Porosity parameters and element composition by X-ray photoelectron spectroscopy of the carbon samples.

**Sample**	**Porosity parameters**					**Elemental composition by X-ray photoelectron spectroscopy (at%)**		
	**${\text{S}}_{\text{BET}}^{1}$ (m^**2**^ g^**−1**^)**	**${\text{V}}_{\text{T}}^{\text{2}}$(cm^**3**^ g^**−1**^)**	**${\text{S}}_{\text{DFT}}^{3}$ (m^**2**^ g^**−1**^)**	**${\text{V}}_{\text{DFT}}^{3}$ (cm^**3**^ g^**−1**^)**	**Pore width^**4**^ (nm)**	**C**	**O**	**s**
C-Li	76	0.06	82	0.06	0.785	82.6	12.3	5.1
C-Na	198	0.10	224	0.09	<0.7, 0.785	78.5	15.9	5.6
C-K	634	0.26	651	0.23	<0.7, 0.926	87.0	8.2	4.8
C-Cs	1037	0.41	1143	0.38	<0.7, 1.007	87.0	8.5	4.5

1 *BET specific surface area determined using N_2_*.

2 *Total pore volume measured at P/P^0^ = 0.9 from the adsorption branch*.

3 *Cumulative QSDFT micropore surface area and pore volume*.

4 *According to the QSDFT pore size distributions in Figure 3B*.

**Figure 4:**
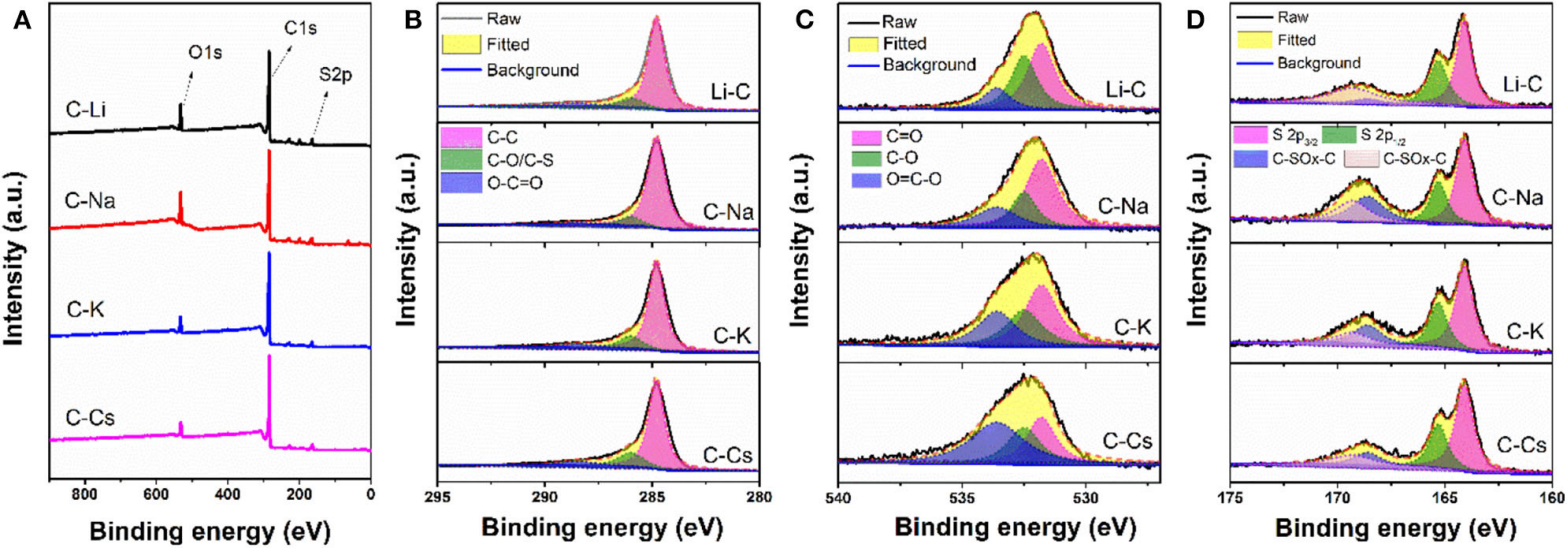
X-ray photoelectron spectroscopy conveys **(A)**, C1s **(B)**, O1s **(C)**, and S2p **(D)** spectra for C-Ms.

To investigate the surface properties of alkali metal ion-activated carbons, X-ray photoelectron spectroscopy analyses are conducted for all the samples (Figure 4). Apart from carbon and oxygen, sulfur could also be observed, suggesting the effective doping of sulfur with a sulfur source of 3-hydroxythiophenol. No metal signal was found in Figure 4A, indicating the removal of metal species due to post-acid washing. As tabulated in Table 1, C-Na and C-Li have obviously higher oxygen content than that of C-K and C-Cs. Combining the result of TGA and SEM images, such high oxygen composition is probably due to the relative lower activation ability of Li^+^ and Na^+^ compared with K^+^ and Cs^+^. C1s spectra can be deconvolved into three peaks, at 284.8, 286, and 288.5 eV, assigning to C-C in aromatic rings, C-O/C-S in alkoxyl/epoxyl/thioether/thiophene, and O-C=O in ester/carboxylic groups, respectively. Oxygen species can be confirmed at 531.8, 532.5, and 533.6 eV, assigning to C=O, C-O, and O-C=O, respectively (Figueiredo et al., 1999). S2p spectra can be deconvolved into four peaks at 164.1, 165.3, 168.6, and 169.3 V. The former two peaks assign to C-S-C species, whereas the latter two peaks assign to oxidized sulfur species (Si et al., 2013; Yu and Park, 2014). Peak assignment results are in Figure 4, and Table 2 reveals that abundant oxygen and sulfur functionalities are presented in all samples. Such heteroatom functionalities can potentially bring about extra pseudocapacitance by reacting with electrolyte. Moreover, heteroatom functionalities on the surface can increase the hydrophilicity of the carbon surface, thus accommodating more electrolyte ions during the energy storage process.

**Table 2:** Peak assignment of C1s, O1s, and S2p for the prepared carbons.

**Peak**	**Binding energy (eV)**	**Assignment**	**Fraction of species (%)**			
			**C-Li**	**C-Na**	**C-K**	**C-Cs**
C1s	284.8	C-C	78.8	85.0	74.8	71.7
	286	C-O/C-S	12.1	10.4	15.6	18.7
	288.5	O-C=O	9.1	4.6	9.6	9.6
O1s	531.8	C=O	49.1	38.6	49.1	26.5
	532.5	C-O	35.1	18.0	35.1	30.3
	533.6	O=C-O	15.8	43.4	15.8	43.2
S2p	164.1	S2p3/2	44.8	38.0	44.8	49.0
	165.3	S2p1/2	27.4	20.6	30.4	25.0
	168.6	C-SOx-C	3.9	19.4	4.0	16.0
	169.3	C-SOx-C	23.9	22.0	23.8	10.0

### Supercapacitive Performance for C-Ms

**Figure 5:**
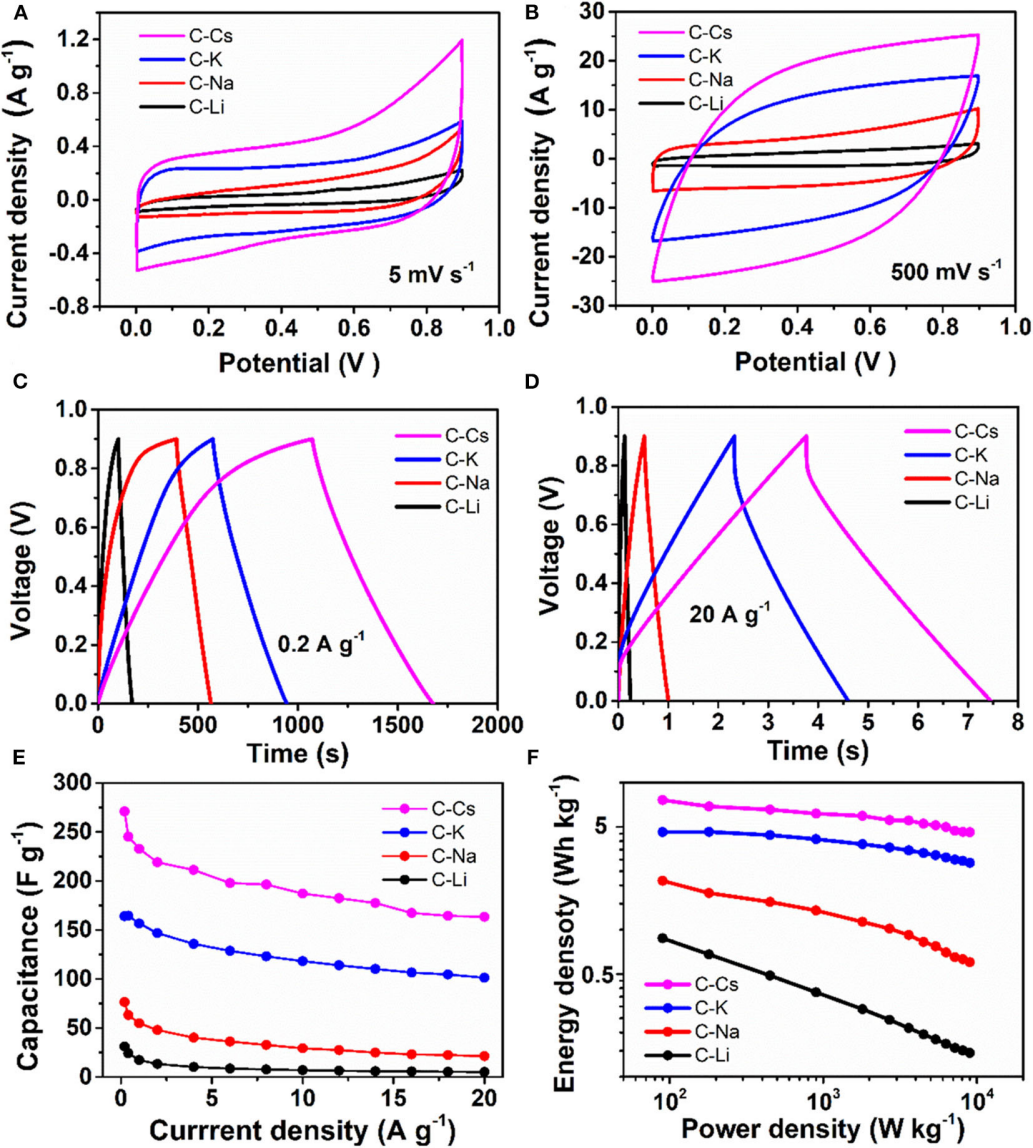
Supercapacitive properties for C-Ms: **(A,B)** Cyclic voltammograms for at scan rates of 5 and 500 mV s^−1^; **(C,D)** galvanostatic potential curves at a current density of 0.2 and 20 A g^−1^; **(E)** Specific capacitance at different current densities; **(F)** Ragone plot.

The symmetrical supercapacitor is assembled with two same electrodes and characterized by cyclic voltammetry and galvanostatic charge–discharge measurements. The CVs at a scan rate of 5 mV s^−1^ show that a rectangular-like CV could be observed for C-K (Figure 5A), suggesting an energy storage mechanism of EDL capacitance. By contrast, there is a deviation from the rectangular-like shape CVs for C-Li and C-Na, especially within the potential range of 0.3–0.9 V, indicating a combined energy storage mechanism of pseudocapacitance together with EDL capacitance (Gu et al., 2013; Wu et al., 2014, 2015). The generated pseudocapacitance is attributed to the O, S functionalities, which can react with an electrolyte based on the proposed redox mechanisms (Sun et al., 2011; Zhao et al., 2012).

$$\begin{array}{c} -{\text{C}}_{\text{x}}\text{O}+{\text{e}}^{-}+{\text{K}}^{+}↔{\text{C}}_{\text{x}}-\text{OK}\\ -\text{S}{\text{O}}_{2}-+2{\text{e}}^{-}+{\text{H}}_{2}\text{O}↔-\text{SO}-+\;2\text{O}{\text{H}}^{-}\\ -\text{SO}-+{\text{e}}^{-}+{\text{H}}_{2}\text{O}↔-\text{S}\left(\text{OH}\right)-+\text{O}{\text{H}}^{-} \end{array}$$

The charge branch for C-Cs shows increased response current densities within the potential range of 0.7–0.9 V, which is probably due to the insertion of ions into the inner pores of C-Cs. As measured by nitrogen sorption analysis, such variation is probably due to the presence of numerous micropores in C-Cs. However, the CVs for all samples at a scan rate of 500 mV s^−1^ show a good symmetry property, especially for C-K and C-Cs, indicating a perfect pore connection in these samples (Figure 5B). The galvanostatic charge–discharge measurements (Figures 5C,D) at low current density (0.2 A g^−1^) show that all of the discharge branches for C-Ms are linear shape, suggesting the EDL capacitance-dominated energy storage mechanism for all samples. The charge branches for C-Na, C-K, and C-Cs present a non-linear characteristic, especially in the potential range of 0.7–0.9 V due to the insertion of ions into the inner pores, corresponding well with the CVs in Figure 5A. The duration of the discharge branch shows a positive correlation with the surface area even at a higher current density of 20 A g^−1^ (Figure 5D). The specific capacitances at each current density for all samples obey the following order: C-Cs > C-K > C-Na > C-Li (Figure 5E). As tabulated in Table 3, the capacitance for C-Cs at 0.2 A g^−1^ could reach up to 270.9 F g^−1^, which is much higher than that of C-K, C-Na, and C-Li. Apart from the high specific capacitance, the capacitance retention ratio as the current density increases from 0.2 to 20 A g^−1^ for C-Cs is 60.3 %, which is close to that of C-K (61.7 %) and much higher than that of C-Na (27.9 %) and C-Li (16.7 %). Combining the results of structural analysis, such a good capacitance retention ratio for C-Cs is possibly due to the high surface area, interconnected pore structure, and heteroatom doping in C-Cs and C-K. The high surface area and interconnected pores can provide a high interface for electrolyte, whereas the heteroatom doping can increase the wettability of carbon surface to an electrolyte, thus accommodating more electrolytes on the interface to generate high capacitance even under high current densities.

**Table 3:** Specific capacitances at different current densities for metal ion activated carbons.

**Sample**	**C**_**m**_ **[F g**^**−1**^**]**^**1**^		**C**_**BET**_ **[μF cm**^**−2**^**]**^**2**^		**Heteroatom content detected by X-ray photoelectron spectroscopy (at%)**	
	**0.2 A g^**−1**^**	**20 A g^**−1**^**	**0.2 A g^**−1**^**	**20 A g^**−1**^**	**O**	**S**
C-Li	31.1	5.2	40.92	6.84	12.3	5.1
C-Na	76.4	21.3	38.59	10.76	15.9	5.6
C-K	164.0	101.2	25.87	15.96	8.2	4.8
C-Cs	270.9	163.4	26.12	15.76	8.5	4.5
KAC^3^	205	-	22.85	-	5.2	-
CsAC^3^	221	162	16.84	12.35	8.2	-

1 *Specific gravimetric capacitance at different current densities*.

2 *Areal capacitance calculated by C_m_/S_BET_*.

3 *BET specific surface areas for KAC and CsAC are 897 and 1,312 m^2^ g^−1^, respectively (Zhou et al., 2016)*.

The impact of heteroatom on the capacitive property is also investigated in our work. The areal capacitances calculated from BET specific surface area are tabulated in Table 3. C_BET_ values for C-Li and C-Na are 40.92 and 38.59 μF cm^−2^, respectively, much higher than that of C-K and C-Cs. The low areal capacitance for C-K and C-Cs can be attributed to the lower content of heteroatom functionalities, which brings about pseudocapacitance for C-Ms. This result could coincide well with the CV analysis, suggesting the presence of significant pseudocapacitance generated by heteroatoms in C-Na and C-Li. As for C-K and C-Cs, their areal capacitances are higher than those reported of KAC and CsAC with a similar preparation method. Such higher areal capacitance could be attributed to the extra sulfur doping in C-K and C-Cs. Moreover, the areal capacitance of C-Cs (15.76 μF cm^−2^) at high current density (20 A g^−1^) is still higher than that of CsAC (12.35 μF cm^−2^), as CsAC has a higher carbon content, which probably contributes to a better conductivity in comparison with C-Cs. The lower areal capacitance for CsAC is attributed to its absence of sulfur functionalities, which can improve the hydrophilicity for porous carbon and accommodate more electrolyte ions even at high current densities. With high specific surface area, well-interconnected porous structure, and oxygen and sulfur doping for C-Cs, supercapacitor cells assembled by C-Cs can deliver an energy density of 7.6 Wh kg^−1^ (Figure 5F), which is much larger than that of other samples.

## Conclusions

Sulfur-doped porous carbon activated by alkali metal ions is prepared through an alkali metal ion activation method in this paper. Such a preparation method with alkali metal ions uniformly anchored in the sulfur-containing resin can realize the high-effective activation by generating interconnected porosity and concentrated pore size distribution. Porous carbon shows an increased surface area as the increasing of adopted metal ions' radius. On the other hand, luxuriant surface oxygen/sulfur functionalities inherited from carbon precursor in porous carbon can effectively improve the hydrophilicity of carbon surface to electrolyte and bring about extra pseudocapacitance. In this case, supercapacitor assembled with C-Cs can deliver an energy density of 7.6 Wh kg^−1^. Our research proposes an effective strategy on the design of porous and surface structure for carbon-based energy storage materials.

## Data Availability Statement

All datasets presented in this study are included in the article.

## Author Contributions

CW did all the work containing experiments, data and manuscript. The author confirms being the sole contributor of this work and has approved it for publication.

## Conflict of Interest

The author declares that the research was conducted in the absence of any commercial or financial relationships that could be construed as a potential conflict of interest.
